# Biochemical verification of tobacco use in ICU patients using point-of-care urinary cotinine

**DOI:** 10.1016/j.ccrj.2026.100185

**Published:** 2026-05-11

**Authors:** Stephen J. Surace, Jeffrey Presneill, Melissa J. Ankravs, Haustine Panganiban, Cara Moore, Alice Barrese, Adam Deane, Mark P. Plummer

**Affiliations:** a Intensive Care Unit, Royal Melbourne Hospital, VIC, Australia; b Department of Critical Care, University of Melbourne, Melbourne, VIC, Australia; c Intensive Care Unit, Royal Adelaide Hospital, SA, Australia; d School of Medicine, University of Adelaide, SA, Australia

**Keywords:** Cotinine, Nicotine, Smoking, Intensive care unit (ICU)

## Abstract

**Objectives:**

Obtaining an accurate smoking history can be challenging in critically ill patients. The objective of this study was to determine the prevalence of nicotine exposure using a point-of-care urine cotinine test in a heterogeneous cohort of critically ill patients and to compare this to the smoking history in the medical record.

**Design, setting, and participants:**

In 481 adult patients we prospectively undertook a urine cotinine test within 24 h of admission using a point-of-care binary colorimetric assay with a detection threshold of 200 ng/mL. Smoking history was retrieved from the electronic medical record.

**Main outcome measures and results:**

Urinary cotinine was detected in 151 of 481 patients (31.4%), exceeding the 134 participants with a documented history of current smoking by 3.5% (relative increase 12.7%). A positive urine cotinine test was reported in 12% (40/347) of participants not known to be actively smoking, including 18 ‘former smokers’, 12 ‘non-smokers’, and 10 in whom smoking status was unknown. Cotinine-positive participants were ventilated for longer than cotinine-negative patients; a median of 71 h vs. 44 h (median difference of 30h, 95% CrI: 5.6 to 56); they were more likely to have a “code-grey” event; 34 of 151 (23%) vs. 24/330 (7.3%); median relative risk of 3.1 (95% CrI, 1.9–5.1); and were more likely to “discharge against medical advice”; 12/125 (10%) vs 3/275 (1%), with a median relative risk of 9.7 (95% CrI, 3.0–46).

**Conclusions:**

Urinary cotinine testing offers a point-of-care determination of current tobacco smoking status in the critically ill.

## 1 Introduction

Globally, over 1 billion people smoke cigarettes.^1^ Smoking remains a major international public health threat, with a global attributable mortality of eight million deaths per year, primarily from ischaemic heart disease, chronic obstructive pulmonary disease, cancer, and stroke.^1^ In Australia, approximately 11% of the adult population are active smokers.^2^ Alarmingly, up to two-thirds of future deaths in this population will be accelerated by smoking.^3^ It is not surprising that smoking is over-represented in critically ill patients.^4^^,^^5^

As well as increasing the risk of chronic disease, smoking modifies the physiological response to acute illness, independently increasing morbidity and mortality in the critically ill patients.^6^ Smoking increases the risk of mechanical ventilation and acute respiratory distress syndrome,^7^ prolongs ICU length of stay, and dose-dependently increases mortality.^6^

An accurate smoking history in the critically ill patient is important for risk stratification and clinical diagnosis, as well as to guide requirement for nicotine replacement therapy.^8^ However, obtaining a self-reported smoking history in this population is often challenging or impossible due to altered levels of consciousness or endotracheal intubation. A collateral smoking history from the next of kin may be unavailable or inaccurate, particularly when estimating quantitative smoking measures (packs per day and years smoked).[9], [10], [11] Consequently, underreporting of smoking in critically ill patients may occur frequently.

Cotinine is a metabolite of nicotine with a long half life of approximately 20 h. It can be detected in blood, urine, saliva, or hair samples making cotinine an ideal biomarker of smoking exposure.^10^^,^^12^^,^^13^ Plasma levels of cotinine and other plasma biomarkers of tobacco exposure have been used in observational studies in critically ill patients with acute respiratory distress syndrome to identify associations with adverse outcomes.^7^^,^^14^ The concentration of cotinine in the urine is higher than in blood or saliva and strongly correlates with the degree of nicotine exposure.^15^ Urine cotinine has been used extensively to distinguish active and passive smokers from non-smokers in epidemiological studies,[15], [16], [17] pregnancy,^18^^,^^19^ childhood asthma,^20^ and peri-transplant,^21^ and to characterise occupational and environmental exposure to tobacco.[22], [23], [24], [25] Clinically, urine cotinine is used to monitor compliance in smoking cessation programs^26^^,^^27^ and in occupational health insurance claims.

Despite familiarity with the widely available test, established cut-off thresholds, ease of use, and low-cost (<$4.00 AUD), there is a paucity of studies assessing urine cotinine in the critically ill. Data are limited to a single-centre observational study of 60 patients admitted to a Californian ICU in which a high proportion of biochemically verified nicotine exposure with urine cotinine was reported in patients deemed ‘non-smokers’ from the medical history.^10^

Accordingly, in a large cohort of critically ill patients in Australia, we aimed to determine the prevalence of positive urine cotinine tests within 24 h of admission, and to compare this to the smoking history documented in the medical record. Secondary aims were to explore associations between biochemically verified nicotine exposure and use of nicotine replacement therapy, behavioural disturbance necessitating security assistance, mechanical ventilation, ICU length of stay, and mortality.

## 2 Methods

This observational cohort study used convenience sampling to recruit adult patients admitted to the Royal Melbourne Hospital Intensive Care Unit with an available urine sample between August 2021 and May 2023. Recruitment ceased when the test kits met their expiry date. Patients were excluded if they were anuric or if a urine sample was not available in the first 24 h of ICU admission. The study was approved by the Royal Melbourne Hospital Office for Research as a quality assurance activity (QA2021046) that did not require formal ethics review and the need for informed consent was waived. The manuscript was prepared in accordance with the Strengthening the Reporting of Observational Studies in Epidemiology guidelines for reporting observational research.^28^

A urine sample was collected within 24 h of admission to the ICU and tested at the bedside with a commercially available urinary cotinine colorimetric test (ScreenClear™, Prometheus, Hangzhou, China) with a detection threshold of ≥200 ng/mL. This method and point-of-care test complies with the Australian standard relevant to specimen collection and the detection of drugs of abuse in urine samples (Urine AS4308:2008). Study data were extracted from the electronic medical record (EMR) including patient demographics, admission type, diagnosis and illness severity (Acute Physiology And Chronic Health Evaluation II score), smoking status [‘current’, ‘former’, ‘non-smoker’ or ‘unknown’], nicotine replacement therapy prescription, duration of mechanical ventilation, “code grey” hospital security attendance events in ICU,^29^ ICU and hospital length of stay, discharge destination, and mortality. Smoking status is recorded as an optional, rather than mandatory, field in the electronic medical record at admission. Consistent with the pragmatic study design, no additional training or changes to routine clinical practice were implemented to influence how smoking history was assessed or documented.

Selected patient demographics and baseline variables were reported as summary statistics. Possible relationships between variables of interest were explored in classification tables, returning estimates of sensitivity, specificity, and positive and negative predictive values. Subsequently, several simple univariable Bayesian regression models were used to examine associations between variables.^30^^,^^31^ For binary outcomes, generalised linear regression models were generated, with results presented as median relative risk (RR), alongside 95% equal-tailed credible intervals indicating the range of values most consistent with the observed data. For continuous outcomes, we used quantile regression models to estimate differences in the median values between groups. These models used neutral prior distributions for all regression coefficients (normal, mean zero, and variance 10,000; with an igamma^1^^,^^1^ prior distribution for variance in the quantile models) so that the results were controlled by the observed data. Models were simulated using four independent Markov chains, each of 30,000 iterations, after having discarded an initial 2500 warm-up (burn-in) iterations to ensure chain stabilisation. “Burn-in” refers to the initial portion of a Markov chain that is not stationary and is still affected by its initial value. Model convergence and fit were confirmed by Gelman–Rubin statistics approaching 1.0, supported by standard graphical and diagnostic assessments. Analyses were performed using Stata version 19.5 (StataCorp, College Station, Texas 77845, USA).

## 3 Results

### 3.1 Patient characteristics

**Fig. 1:**
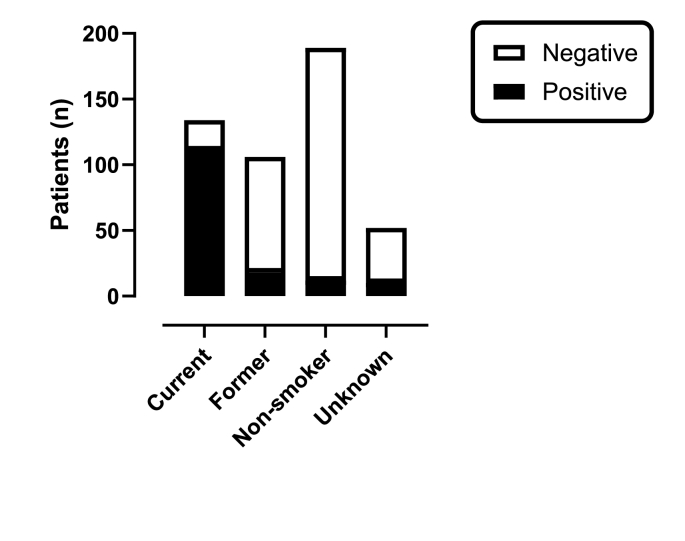
Urine cotinine according to clinical smoking category.

The study recruited 481 participants with baseline characteristics shown in Table 1 according to their documented smoking status category. There were 151 participants (31%) with a positive urinary cotinine test, exceeding the 134 documented ‘current smokers’ (3.5% absolute difference and 12.7% relative difference). Of those documented as ‘current smokers’, 83% (111/134) had a positive urine cotinine test, whereas 6% (12/189) of ‘non-smokers’ had a positive urine cotinine test. In addition, 17% (18/106) of ‘former smokers’ and 19% (10/52) of those with unknown smoking status had a positive urine cotinine test (Table 2 and Fig. 1). Accordingly, a urine cotinine test identified recent nicotine exposure in 12% (40/347) of participants not known to be actively smoking or where smoking status was unknown.

**Table 1:** Selected patient characteristics according to smoking history.

		Current	Former	Nonsmoker	Unknown	Total N (col %) or mean (SD)
All subjects	n (row %)	134 (28)	106 (22)	189 (39)	52 (11)	481 (100)
Female		42 (23)	27 (15)	87 (49)	23 (13)	179 (37)
Male		92 (30)	79 (26)	102 (34)	29 (10)	302 (63)
Age, y, all	Mean (SD)	50 (16)	63 (14)	55 (18)	53 (23)	55 (18)
Female		52 (17)	56 (14)	59 (17)	60 (23)	57 (18)
Male		49 (15)	65 (13)	52 (19)	48 (23)	54 (18)
APACHE II	Mean (SD)	8.7 (4.1)	10 (4.7)	8.4 (3.9)	10 (5.4)	9.1 (4.4)
Admission type	n (row %)					
Planned		26 (20)	48 (36)	49 (37)	9 (7)	132 (27)
Unplanned		108 (31)	58 (17)	140 (40)	43 (12)	349 (73)
Mech vent	n (row %)					
No		35 (19)	52 (28)	91 (49)	7 (4)	185 (38)
Yes		99 (33)	54 (18)	98 (33)	45 (15)	296 (62)
Referral source	n (row %)					
ED		85 (34)	36 (14)	91 (36)	40 (16)	252 (52)
Other Hosp		26 (33)	8 (10)	33 (42)	11 (14)	78 (16)
Post Op		22 (17)	51 (38)	59 (44)	1 (1)	133 (28)
Ward		1 (6)	11 (61)	6 (33)	0	18 (4)

APACHE II: Acute Physiology And Chronic Health Evaluation core version II; ED: emergency department; Mech vent: mechanical ventilation; Other Hosp: other hospital; SD: standard deviation.

Post Op: postoperative; Ward: hospital ward.

**Table 2:** Smoking status compared to positive cotinine results overall and in the subgroup not receiving nicotine replacement therapy.

Smoking status	Cotinine		Total N (col %)
n (row %)	Negative	Positive	
Current	23 (17)	111 (83)	134 (28)
No nicotine replacement	17	72	89
Former	88 (83)	18 (17)	106 (22)
No nicotine replacement	87	17	104
Non-smoker	177 (94)	12 (6)	189 (39)
No nicotine replacement	177	12	189
Unknown	42 (81)	10 (19)	52 (11)
No nicotine replacement	42	10	52
Total	330 (69)	151 (31)	481 (100)
No nicotine replacement	323	111	434

When assessed as a dichotomy, the clinical classification of ‘current smoker’ versus a combination of the other three categories ‘former’, ‘unknown’, and ‘non-smoker’ predicted a positive cotinine test with a sensitivity of 74%, specificity 93%, positive predictive value of 83%, and a negative predictive value of 88%. Further evaluation of this relationship between clinical and chemical diagnoses of current smoking returned a median RR estimate (with 95% equal-tailed credible intervals, CrI) of 13[8], [9], [10], [11], [12], [13], [14], [15], [16], [17], [18], [19], [20], [21], [22], [23], [24], [25] for a positive cotinine test among documented ‘current smokers’ compared to the binary alternative. Other RR estimates were 2.7 (1.3–5.6) and 3.0 (1.3–6.5), respectively, for patients in the clinical categories of ‘former smoker’ and ‘unknown’ smoking status.

### 3.2 Sensitivity analysis based on nicotine replacement therapy prescription

Nicotine replacement therapy was used in 34% (45/134) of ‘current smokers’ and 2% (2/106) of ‘former smokers’. Nicotine replacement therapy was not prescribed to any participants with ‘unknown’ or ‘non-smoker’ status (Table 2).

As a sensitivity analysis for the possibility of confounding of urine cotinine tests by nicotine replacement therapy, the above regression analyses were repeated in the smaller cohort without nicotine replacement therapy. Binary status as a ‘current smoker’ predicted a positive cotinine test with a somewhat diminished sensitivity of 65% but preserved specificity (95%), positive predictive value (81%) and negative predictive value (88%). The Bayesian binary regression returned an unchanged median RR (95% CrI) estimate of 13[8], [9], [10], [11], [12], [13], [14], [15], [16], [17], [18], [19], [20], [21], [22], [23], [24], [25] for a positive cotinine test among documented ‘current smokers’, excluding nicotine replacement therapy, compared to the alternative. The other estimates, 2.6 (1.3–5.5) and 3.0 (1.3–6.7), respectively, for patients in the clinical categories of ‘former smoker’ and ‘unknown’ smoking status, were also minimally changed.

### 3.3 Associations between positive urine cotinine and outcomes

Overall, 62% (296/481) of participants were mechanically ventilated. Cotinine-positive participants were more likely to receive mechanical ventilation; median RR 1.3 (95% CrI, 1.1–1.5). If ventilated, cotinine-positive participants were ventilated for longer than cotinine-negative patients; median 71 h vs. 44 h; (median difference 30h, 95% CrI: 5.6 to 56). Regardless of ventilation status, there was also an increase in the median overall ICU admission duration with cotinine-positive versus cotinine-negative patients (a median of 3.2 days vs. 2.3 days and a median difference of 0.8 days, 95% CrI: 0.1 to 1.6). However, the overall median hospital length of stay (cotinine-positive 7.4 days vs. continue-negative 7.3 days) was not substantially different between groups.

Behaviour disturbance triggering a “code grey” hospital security response in ICU was observed in 12% (58/481) of participants. Cotinine-positive participants were more likely to have a “code-grey” event than those with a negative urine cotinine test; 34/151 (23%) vs. 24/330 (7.3%); median RR, 3.1 (95% CrI, 1.9–5.1).

Hospital mortality was 17% (80/481), with no difference observed between groups. Cotinine-positive participants who survived hospital were more likely to “discharge against medical advice”; 12 out of 125 (10%) vs 3 out of 275 (1%), Table 3; median RR, 9.7 (95% CrI, 3.0–46).

**Table 3:** Discharge destinations for hospital survivors according to cotinine detection status.

Destination	Cotinine		Total N (column %)
n (column %)	Negative	Positive	
Home	192 (70)	81 (65)	273 (68)
Other acute hospital	39 (14)	18 (14)	57 (14)
Rehabilitation	40 (14)	13 (10)	53 (13)
Discharge against medical advice	3 (1)	12 (10)	15 (4)
Care facility	1 (0.4)	1 (0.8)	2 (0.5)
Custody	1 (0.4)	0	2 (0.5)
Total	276 (100)	125 (100)	401 (100)

## 4 Discussion

In this large cohort of critically ill adults, point-of-care urinary cotinine testing identified recent nicotine exposure in nearly one-third of patients, a prevalence exceeding the proportion documented as current smokers in the medical record. Importantly, 12% of patients classified as former smokers, never smokers, or with unknown smoking status had biochemical evidence of nicotine exposure. These findings highlight a substantial discordance between clinical documentation and biochemical evidence of nicotine exposure and underscore the limitations of relying solely on a smoking history in the ICU. These data also support established associations between recent nicotine exposure and adverse outcomes, including higher likelihood of mechanical ventilation, longer duration of ventilation and ICU stay, and an increased incidence of behavioural disturbance requiring security intervention.[9], [10], [11]^,^^32^ We also report a new finding of increased rates of discharge against medical advice in cotinine-positive patients.

Difficulties obtaining an accurate smoking history in the ICU are well recognised owing to impaired consciousness, mechanical ventilation, communication barriers, and incomplete collateral history. Observational studies consistently report a high degree of misclassification of smoking status in critically ill patients when relying on history alone.[9], [10], [11] In a single-centre study of 60 critically ill patients, Hsieh et al. found that biomarkers including serum and urine cotinine, trans-3′-hydroxycotinine, and 4-methylnitrosamino-1-3-pyridyl-1butanol (NNAL) identified active smoking in 21% of patients labelled as ‘non-smokers’ and passive smoke exposure in an additional 75%.^10^ Having identified NNAL as being highly specific for tobacco smoking, this group then undertook a larger observational study analysing urinary NNAL in 381 participants with Acute Respiratory Distress Syndrome (ARDS) from across the United States.^14^ They report similar levels of discordance to our study, with urinary NNAL classifying 9% of reported ‘non-smokers’ and 44% of those with ‘unknown’ smoking history as active smokers.^14^ Despite being younger and having fewer comorbidities, biomarker-positive smokers exhibited similar severity of lung injury to non-smokers, suggesting increased biological susceptibility to ARDS.^14^ The association between active smoking and risk of ARDS was further supported by a prospective cohort study of 635 patients with blunt trauma in which active and passive smoke exposure, quantified by plasma cotinine, increased the risk of ARDS (adjusted odds ratio of 1.9 and 2.6, respectively).^7^ Notably, low levels of plasma cotinine consistent with passive smoking conferred the greatest risk suggesting a threshold effect rather than a dose–response relationship.^7^ These findings emphasise that unrecognised smoking exposure carries physiological consequences relevant to ICU outcomes and reinforces the importance of accurate detection. Although our cohort study was not focused on ARDS, the increased incidence and duration of mechanical ventilation exposure parallels the signal for smoking-related harm from these multicentre studies.

Collectively, these studies illustrate that biochemical verification consistently reveals an important higher rate of nicotine exposure relative to the documented smoking history, whether for active or passive smoking, and across heterogeneous ICU populations. The present study expands this evidence base by demonstrating the feasibility and clinical relevance of an affordable (<$4.00 AUD) point-of-care test in a large general ICU cohort.

### 4.1 Clinical implications

These data identify that the rate of smoking in adult critically ill patients is almost threefold higher than the background adult rate in Australia.^2^ Accurate identification of nicotine exposure has several implications for ICU management. First, nicotine withdrawal can precipitate agitation, delirium, and behavioural disturbances.^32^ We observed a threefold increase in “code grey” events among cotinine-positive patients, suggesting that undetected nicotine dependence may contribute to ICU behavioural risk. Targeted use of nicotine replacement therapy may mitigate such episodes and reduce the need for sedatives, restraints, and security intervention. Second, nicotine and tobacco smoke constituents have potent physiological effects including increased sympathetic activation, impaired mucociliary clearance, endothelial dysfunction, platelet activation, and increased oxidative stress.[33], [34], [35], [36] These pathways plausibly contribute to the higher likelihood of mechanical ventilation, prolonged ventilation duration, and longer ICU stay among cotinine-positive patients in our study. Appreciation of this risk on admission is important for routine clinical care. Third, unrecognised smoking status limits clinicians’ ability to provide targeted smoking cessation interventions, which may be especially impactful during acute hospitalisation, a period regarded as a “teachable moment”.[37], [38], [39], [40] Biochemical detection thus supports both personalised clinical management and broader public health objectives. Finally, for epidemiological and interventional studies, precise exposure classification is essential. Misclassification of smoking status introduces residual confounding and may distort risk estimates in studies of respiratory failure, sepsis, and perioperative outcomes.^41^ A rapid, affordable bedside test may therefore strengthen observational research by improving covariate accuracy.

Several limitations should be acknowledged. First, the point-of-care assay provides only a binary threshold (≥200 ng/mL) and cannot quantify smoking intensity, pack-years, or distinguish active from passive exposure. Second, cotinine reflects nicotine intake from any source; we were unable to assess pre-hospital use of nicotine replacement therapy, vaping, e-cigarettes, tobacco-laced cannabis, or smokeless tobacco. Although our sensitivity analysis excluding nicotine replacement therapy prescription in ICU produced similar estimates, some cotinine-positive results may reflect noncombustible nicotine use. Third, urinary cotinine concentration is influenced by urine dilution and renal function.^42^ Unlike NNAL, cotinine is more susceptible to variability related to creatinine concentration, and we did not perform creatinine correction. Fourth, the observational design precludes causal inference, and residual confounding, particularly from socioeconomic factors, substance use, and mental health disorders cannot be excluded.^43^ Fifth, the single-centre design limits external validity; however, it is reassuring that the rate of “current smoking” is similar to that reported in other Australian centres.^40^ Finally, while the urine cotinine assay meets Australian standards for drug detection, test validation data were unavailable. While comparable lateral flow immunoassays demonstrate high sensitivity, their specificity varies according to the cotinine threshold applied, introducing the potential for misclassification of smoking status, particularly among individuals with low-level nicotine exposure.^17^^,^^44^

## 5 Conclusions

Biochemical verification using an affordable point-of-care urinary cotinine test revealed substantially higher nicotine exposure than documented smoking histories from the clinical record. These findings mirror prior biomarker-based studies in critical illness and highlight the clinical and epidemiological value of objective exposure assessment. Future studies should include a health economics analysis to evaluate the integration of rapid cotinine testing into ICU workflows and assess its potential to inform clinical decision-making and smoking cessation strategies.

## CRediT authorship contribution statement

**SS:** Data Curation, Writing – Original Draft, Funding Acquisition. **JP:** Formal Analysis, Writing – Review and Editing. **MA:** Resources, Project Administration, Review and Editing. **HP:** Software, data collection resources, Review and Editing. **CM:** Project Administration, Review and Editing. **AB:** Investigation coordination, data collection, Review and Editing. **AD:** Resources, Supervision, Review and Editing. **MP** Conceptualisation, Methodology, Resources, Writing – Review and Editing, Supervision.

## Data availability statement

De-identified data are available by request from the corresponding author.

## Declaration of competing interest

The authors declare the following financial interests/personal relationships which may be considered as potential competing interests: Stephen Surace reports financial support was provided by Intensive Care Foundation. Associate Professor Mark Plummer and Professor Adam Deane are on the Editorial board of Critical Care and Resuscitation. Given their role as editorial board members, they had no involvement in the peer review of this article and had no access to information regarding its peer review. Full responsibility for the editorial process for this article was delegated to another journal editor. If there are other authors, they declare that they have no known competing financial interests or personal relationships that could have appeared to influence the work reported in this paper.
